# Analytical approaches to detect maternal/fetal genotype incompatibilities that increase risk of pre-eclampsia

**DOI:** 10.1186/1471-2350-9-60

**Published:** 2008-07-03

**Authors:** Neeta Parimi, Gerard Tromp, Helena Kuivaniemi, Jyh Kae Nien, Ricardo Gomez, Roberto Romero, Katrina AB Goddard

**Affiliations:** 1 Department of Epidemiology and Biostatistics, Case Western Reserve University, Cleveland, OH, USA; 2 Center for Molecular Medicine and Genetics, Wayne State University, Detroit, MI, USA; 3 Department of Neurology, Wayne State University, Detroit, MI, USA; 4 Department of Surgery, Wayne State University, Detroit, MI, USA; 5 the Perinatology Research Branch, NICHD, NIH, Bethesda, MD, USA; 6 Center for Perinatal Diagnosis and Research, Sotero del Rio Hospital, Pontificia Universidad Catolica de Chile, Santiago, Chile; 7 Center for Health Research, Kaiser Permanente Northwest, 3800 N. Interstate Avenue, Portland, USA

## Abstract

**Background:**

In utero interactions between incompatible maternal and fetal genotypes are a potential mechanism for the onset or progression of pregnancy related diseases such as pre-eclampsia (PE). However, the optimal analytical approach and study design for evaluating incompatible maternal/offspring genotype combinations is unclear.

**Methods:**

Using simulation, we estimated the type I error and power of incompatible maternal/offspring genotype models for two analytical approaches: logistic regression used with case-control mother/offspring pairs and the log-linear regression used with case-parent triads. We evaluated a real dataset consisting of maternal/offspring pairs with and without PE for incompatibility effects using the optimal analysis based on the results of the simulation study.

**Results:**

We identified a single coding scheme for the incompatibility effect that was equally or more powerful than all of the alternative analysis models evaluated, regardless of the true underlying model for the incompatibility effect. In addition, the log-linear regression was more powerful than the logistic regression when the heritability was low, and more robust to adjustment for maternal or fetal effects. For the PE data, this analysis revealed three genes, lymphotoxin alpha (*LTA*), von Willebrand factor (*VWF*), and alpha 2 chain of type IV collagen (*COL4A2*) with possible incompatibility effects.

**Conclusion:**

The incompatibility model should be evaluated for complications of pregnancy, such as PE, where the genotypes of two individuals may contribute to the presence of disease.

## Background

Typically, many diseases or complications arising during pregnancy, such as pre-eclampsia (PE), have been considered primarily maternal disorders. For example, in the context of genetic studies, it is frequently the maternal genotype that is considered in evaluating increased risk for disease (summarized in [1]). However, this narrow view ignores potential contributions from the fetal genome or other mechanisms of disease, such as genomic conflict [2]. For example, one of several proposed mechanisms of disease for PE is that the fetus demands a greater blood supply than is beneficial for the mother to provide, resulting in hypertension in the mother [3]. Other potential mechanisms include genomic imprinting, gestational drive, or incompatibility between the maternal and fetal genomes that can lead to diseases such as Rh incompatibility. Thus, it may be critical to evaluate maternal-fetal genetic interactions to fully understand the genetic contributions to many disorders or complications of pregnancy.

In this paper we will focus on the genetic 'incompatibility' between maternal and fetal alleles, i.e., where the maternal and fetal genotypes do not exactly match. One possible explanation for the underlying biological mechanism of disease in this scenario is that the genetic differences between the mother and fetus may induce an immunological response by the mother. One of the best known examples of fetal contributions to risk of pregnancy related complications is a genetic incompatibility between mother and fetus at the RhD locus. RhD hemolytic disease of the newborn (HDN) occurs when the mother is Rh negative (dd) and does not possess the allele for the antigen present in the offspring (Dd), and has been immunized by transplacental passage of RhD-positive red cells during a previous pregnancy [4]. This particular incompatibility presents an adverse prenatal environment where the mother produces antibodies to the D allele present in the fetus. However, it is important to remember that the genetic 'incompatibility' may, in fact, be beneficial rather than harmful, as will be discussed further below. In addition, biological mechanisms aside from an immunological response could produce an incompatibility effect, and thus, alternative models of disease need to be explored. Our terminology differs from that used by other investigators [5-9], in that genotypes are compatible or incompatible depending on whether they are the same or different for the maternal-fetal pair, and the 'incompatible' genotype combinations can have beneficial or adverse effects. In contrast, other investigators define a maternal-fetal incompatibility to be "...a maternal-fetal genotype combination that can adversely affect the developing fetus... [5]." Thus, under this definition the term 'incompatibility' refers only to adverse effects, but can correspond to genotype combinations that are the same or different for the maternal-fetal pair.

PE, a leading cause of maternal and perinatal mortality and morbidity worldwide, affects 3–7% of all pregnancies in developed countries [10-12], although rates may be lower in non-industrialized countries (1.6–5.5%) [13-18]. The etiology and pathophysiology of PE are incompletely understood, but a combination of maternal/fetal genetic predisposition and environmental factors have been implicated as potential risk factors for the disease [10,19].

Some investigators have proposed that PE may be due to an abnormal maternal immune response to a semi-allogenic fetus [19,20]. Hence, it can be hypothesized that genetic models of maternal/fetal interactions, in particular maternal and fetal genotype incompatibility, can offer a possible mechanism of genetic action in PE. Despite the growing epidemiological evidence for the role of fetal (paternal) alleles and their interaction with maternal alleles, the hypothesis of maternal/fetal genotype incompatibility as a potential mechanism of PE has not been well explored. Only a few studies [19,21] have evaluated the maternal/fetal genotype incompatibility model for a 14 base pair (bp) deletion in exon 8 of the HLA-G gene, and no significant evidence for an HLA-G antigen incompatibility between the mother and fetus in PE was observed in cases where the fetus carried the 14 bp deletion that the mother did not carry. However, Hylenius and coworkers [21] did indicate that the risk of PE is higher in mother-offspring HLA-G genotype combinations that share more than one copy of the 14 bp deletion.

In evaluating the incompatibility model, different underlying mechanisms for the genetic incompatibility can be proposed. For example, the incompatibility effect is not necessarily asymmetric, as in the case of the RhD locus, and disease could occur anytime the fetus possesses an allele that is not present in the mother, regardless of which allele it is. Thus, it remains to be investigated what is the best approach for evaluating the incompatibility model when the true underlying genetic model is unknown.

A second consideration is the study design and analytical approach used to evaluate association between incompatible maternal/offspring genotypes and disease risk. We consider two alternatives. The first approach was selected to reflect the study design for the PE study that we evaluate here, where case and control mother/offspring pairs were recruited. For this study design, the case and control pairs can be compared using logistic regression. Alternatively, we consider a second approach using log-linear regression (also called the Maternal Fetal Incompatibility test, as discussed further below), which compares the distribution of genotypes of case parent-offspring trios to their expected distribution. This approach potentially has several advantages in that the paternal contribution to disease can be more fully understood since the paternal genotype is known, and the trio design offers the opportunity to control for population stratification using family-based approaches [22]. We proposed to study these two analytical approaches although were limited to performing the logistic regression for the PE dataset. Studying the two approaches would allow us to determine the potential loss or gain of power attributed to the analytical approach. For simplicity, in the remainder of the paper we will refer to these alternatives as the 'logistic regression model' and the 'log-linear regression' model, keeping in mind that approaches differ not only by the analytical model, but also by the sampling approach and structure of the data.

The log-linear regression model for studying incompatibility effects was first proposed by Sinsheimer and coworkers [5], called the Maternal Fetal incompatibility test (MFG). The MFG test renders independent tests of association between disease risk and incompatible maternal and offspring genotypes. Sinsheimer states that that the MFG test is preferable to the common practice of using contingency tables analysis for testing maternal fetal incompatibilities as risk factors since use of contingency tables can lead to misattributing maternal main effects or fetal main effects to the incompatibility effects. Sinsheimer did not, however, look at the differences between the logistic regression and the log-linear regression analysis. Starr and coworkers [23] conducted a study comparing the power to detect maternal main effects using the logistic regression with maternal fetal case control samples and using log-linear regression with case parent triads. However the study did not consider the MFG incompatibility risk. The study found that the log-linear approach was more powerful than the unconditional logistic approach. In addition, the log-linear approach was more robust than the logistic regression when maternal effects were parameterized but the actual effects were fetal. This has also been previously shown by Wilcox and coworkers [24] that log-linear maternal and fetal main effect tests are orthogonal, and hence tests for maternal main effects are the same whether or not fetal effects are accounted for in the model. However, Sinsheimer et al (2005) [5] showed in their simulations, that introduction of the MFG incompatibility effect destroys the orthogonality between the maternal or fetal main effects and marginal tests of either effect will no longer give the same results as conditional tests that account for the other effect. This suggests that the conclusions of Starr and coworkers of the superiority of the log-linear approach to the logistic approach cannot be immediately assumed when testing the MFG incompatibility effects and hence provides the justification for our study.

To clarify the relative advantages of these two analytical approaches, we conducted a simulation study to evaluate the power and type I error of several defined incompatibility models and their performance using the logistic regression and log-linear regression model unadjusted and adjusted for maternal and fetal effects. The following three a priori hypotheses were tested: i) A single most powerful incompatibility model with good statistical properties (e.g., power and the type I error rate) exists regardless of the true underlying model; ii) The logistic regression has comparable power to the log-linear regression when maternal and fetal effects are absent and unadjusted for in the model; and iii) The log-linear regression performs better than the logistic regression when maternal and fetal effects are present and adjusted for. The best analysis model was used to identify maternal/fetal genotype incompatibilities in the PE dataset using the logistic regression. In this study, we show that a single analytical model exists with the best performance under conditions similar to PE when the true underlying generating model is unknown. In addition, the performance of the logistic regression compared to the log-linear regression is dependent on heritability and on the adjustment of the presence of maternal and fetal effects. Although we were limited to performing the logistic regression in the PE dataset, overall power of the log-linear regression was only 10% greater than the logistic regression.

## Methods

### Incompatibility Models

**Figure 1:**
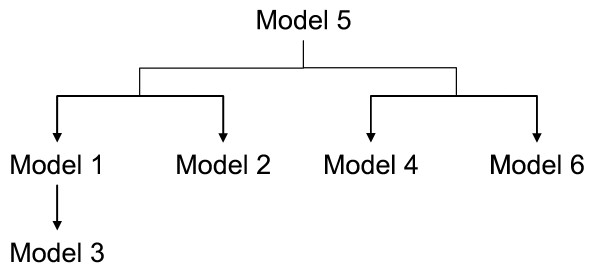
Relationship between biologically plausible models of incompatibility.

A genotype incompatibility arises when the maternal and fetal genotypes are different (Table 1). Several unique biologically plausible models of incompatibility were determined (Table 2) from all the possible maternal/offspring genotype combinations of a two allele locus. The relationship between the models is described in Figure 1, indicating that model 5 encompasses all of the other models in that anytime the maternal and offspring genotypes are different; the pair is coded as having an incompatibility. For example, the RhD locus is an example of Model 3, where the incompatibility effect only occurs if the mother does not possess the rarer allele. This is identical to the incompatibility proposed by the MFG test [5]. Model 3 is nested within the more general model 1, which describes a similar mechanism of disease, where the presence of either allele in the offspring that is not present in the mother could increase susceptibility to disease. Models 4 (or 6) describe a situation where an excess of the "A" (or "a") allele in the mother-offspring pair can have an additive effect on the risk of incompatibility. If the mother offspring pair possess exactly three copies of a specific allele, this combination may increase risk to disease.

**Table 1:** Possible maternal and offspring genotype combinations under Mendelian inheritance

Maternal genotype (g_m_)	Offspring genotype (g_off_)		
	
	AA	Aa	Aa
AA	C_1_	I_1_	-
Aa	I_2_	C_2_	I_3_
aa	-	I_4_	C_3_

C = compatible genotype combinations; I = incompatible genotype combinations

**Table 2:** Biologically plausible models of incompatibility

Model		Maternal Genotype (g_m_)	Offspring Genotype (g_off_)	Scenario
1	I_1_	AA	A**a**	Symmetric Incompatibility: offspring has *any *allele that mother does not
	I_4_	aa	**A**a	
2	I_2_	A**a**	AA	Gestational Drive: Mother has an allele that offspring does not
	I_3_	**A**a	aa	
3	I_1_	AA	A**a**	Asymmetric Incompatibility: offspring has a particular allele ("a") that mother does not
4	I_2_	A**a**	**AA**	Mother-offspring pair possess 3 copies of the "A" allele
	I_1_	**AA**	Aa	
5	I_1_	AA	A**a**	Any difference between maternal and offspring genotypes will result in disease
	I_2_	A**a**	AA	
	I_3_	**A**a	aa	
	I_4_	aa	**A**a	
6	I_3_	A**a**	**aa**	Mother-offspring pair possess 3 copies of the "a" allele
	I_4_	**aa**	A**a**	

### Generation of the Simulated Datasets

Datasets were generated for each incompatibility model (Table 2). One hundred datasets each with sample size of 100 families were randomly generated for each of the incompatibility models. Values of the disease prevalence and heritability were chosen based on previously published studies for PE [11,25,26], and a population prevalence of 5% and broad sense heritability estimates of 0.15, 0.25 and 0.35 were assumed. In addition, a recombination fraction of 0 and linkage disequilibrium parameter of 1 was assumed (i.e., the measured locus is the disease susceptibility locus). For clarity, the model that was used to simulate the data will be referred to as the generating model, while the model used to evaluate the data will be referred to as the analysis model.

The first step in the simulation was to generate genotypes for the parent-offspring trio. Assuming Hardy-Weinberg equilibrium, genotypes of mothers and fathers were generated under allele frequencies for the high-risk allele, *f*, of 0.1 to 0.4 in increments of 0.1. Mendelian inheritance was used to generate the offspring genotype given the parental genotypes. Overall, 100,000 triads were generated for each allele frequency. Next, we simulated the phenotypic value given the genotypes of the trio. In order to adhere to the notion that PE is a disease of pregnancy, the phenotype is not assigned to a particular individual but rather to the mother/offspring pair.

### The Genetic Model

Let P_i _denote the underlying quantitative liability phenotype of the i^th ^family unit in a sample of N families, and g_m_, g_off_, the genotypes of the mother and offspring at the candidate locus, respectively. μ denotes the overall mean of the liability while the polygenic and environmental component E_o_, is a random value from a normal distribution with a specified mean μ_e _and variance σ^2^_e_. X_i _is an indicator variable that represents the genotype combination of the mother/offspring pair, and is 0 if the incompatibility does not exist and is 1 if it does exist. β is the incompatibility parameter that represents the degree of deviation from the overall mean due to the incompatibility effect. The following equation was used to compute the phenotypic value for each mother/offspring pair given known model parameters:

*P*_*i *_= *μ *+ *βX*_*i*_(*G*_*m*_, *G*_*off*_) + *E*_*o*_.

Without loss of generality, the overall mean and environmental mean were restricted to 0. To ensure that the heritability and the prevalence remained at the pre-specified levels, the environmental variance (σ^2^_e_) and incompatibility effect (β) were selected based on these values in addition to the proportion of families with an incompatibility. A random value for the environmental variance (σ^2^_e_) was generated, and the incompatibility effect was computed using the following equations for the locus-specific heritability (h^2^) and total phenotypic variance (σ^2^_T_):

$$h^{2}=1-\frac{\sigma_{e}^{2}}{\sigma_{T}^{2}}$$

$$\sigma_{T}^{2}=\beta^{2}(p)(1-p)+\sigma_{e}^{2},$$

where p is the proportion of incompatible maternal/offspring genotypes in the simulated dataset and is a function of the allele frequency.

To generate cases, a threshold (T) was determined from the model parameters such that 5% of the phenotypic values exceeded the threshold. An additional constraint was that the proportion of mother/offspring pairs that exceeded the threshold (T) given an incompatibility [X_i_(G_m_, G_off_) = 1], was set to approximately 50%. This constraint was added to generate a more complex genetic model in which other causes of disease may exist (either environmental or genetic) aside from the incompatibility. In certain models and under certain allele frequencies, it was not possible to satisfy this constraint given the prevalence of 5%. In such cases the proportion of mother/offspring pairs that exceeded T given an incompatibility was relaxed to fit a prevalence of 5%.

To evaluate type I error, parent-offspring triads were generated by specifying a locus-specific heritability of 0 under each allele frequency, and β was 0. One hundred datasets were simulated for each generating model in addition to the null model of no incompatibility effects. Each dataset was evaluated with all the models listed in Table 2, so the generating model and the analysis model were not necessarily the same. The purpose of using all of the possible analysis models is that for a real dataset, the true underlying generating model is not necessarily known.

### Logistic Regression Model

The datasets for each generating model were analyzed by fitting an unconditional logistic regression model. Although fathers were simulated along with mothers and offspring, the fathers were ignored for the analysis, and the dataset consisted of equal numbers of case and control mother/offspring pairs. Given that the prevalence was constrained to 5%, and 100,000 families were simulated, all of the 5,000 case families were used, and 5,000 control families were randomly selected from the remaining 95,000 non-case families. The 5,000 case and control families were divided into 100 datasets with 50 case and 50 control maternal/offspring pairs each. Hence a total of 200 individuals per dataset were analyzed. The regression was repeated for each of the 100 datasets for each combination of allele frequency and heritability estimate. Models that adjusted for maternal and fetal risk effects, maternal risk effects only, fetal risk effects only or neither maternal nor fetal risk effects in addition to the incompatibility effect were evaluated and these likelihoods are given in the appendix as equations 1–4.

### The Log-linear Regression

The log linear regression was conducted using two different sample sizes. To keep the number of individuals comparable to that used for the logistic regression models, the 5,000 simulated case families were divided into 75 datasets with 67 case parent-offspring trios each. In addition, the analysis was also conducted by keeping the number of families constant by using a sample size of 100 parent-offspring trios. Analogous to the analysis for the logistic regression, we evaluated models unadjusted for maternal risk and fetal risk effects, and adjusted for maternal risk and fetal risk effects, maternal risk effects only and fetal risk effects only, when assessing the incompatibility. The likelihoods are specified in the appendix as equations 5–8.

### Statistical Power and Type I error rate

Statistical power was calculated as the proportion of simulated datasets in which the incompatibility effect was significantly associated with disease at the alpha level of 0.05. Statistical power was calculated for each of the analysis models tested in all of the true generating model datasets simulated with different allele frequencies and heritability values. In addition, power was also calculated for all of the analysis models adjusted or unadjusted for maternal and/or fetal effects.

The type I error rate was calculated similarly, where the error was calculated as the proportion of datasets simulated under the null generating model of no heritability, in which the incompatibility effect was significant at the nominal alpha level of 0.05. Type I error was calculated for all analysis models adjusted and unadjusted for maternal and/or fetal effects under the different allele frequencies.

### The PE Study Population

The study design was described previously [1]. Briefly, subjects were recruited through the Department of Obstetrics and Gynecology at Sotero del Rio Hospital in Puente Alto, Chile at the time of admission to the hospital for delivery, or as part of a longitudinal cohort study to predict obstetrical complications such as PE. The mother provided a blood sample at the time of enrollment, and blood of fetal origin was collected from the umbilical cord following delivery. Clinical and demographic information was obtained by trained personnel using a data collection form. PE was defined as systolic blood pressure ≥ 140 mm/Hg or diastolic blood pressure ≥ 90 mm/Hg on two occasions 6 hours apart, and proteinuria of 300 mg/24 hours or ≥ 2 (dipstick) on two occasions 6 hours apart. For the purposes of this study, women with eclampsia (n = 7) or haemolysis, elevelated liver enzymes low platelet count (HELLP) syndrome (n = 18) were excluded, to limit the possible effects of genetic heterogeneity. Control families were full term and without obstetrical complications including PE, small for gestational age, and placentae previa among others.

The biology or pathophysiology of obstetrical complications including preterm delivery, PE, preterm premature rupture of membranes, and infants who are small for gestational age was used as the basis for selecting the candidate genes in this study. Candidate genes were selected from pathways including immune response, uteroplacental ischemia, and angiogenesis. A complete list of all 190 candidate genes is provided by Goddard and coworkers [1]. SNP discovery within the candidate genes was performed by DNA sequencing at Genaissance Pharmaceuticals, Inc. (New Haven, CT). SNPs were selected for genotyping in order to capture at least 90% of the haplotypic diversity of each gene, as described previously [1]. A total of 775 single nucleotide polymorphisms (SNPs) from 190 genes were analyzed in 324 PE mother/offspring pairs and 602 control mother/offspring pairs after eliminating families with obvious relationship errors and genotyping errors in the SNPs. Important covariates that were identified previously were maternal age, offspring gender, and body mass index (BMI), and were included in all subsequent analyses. No evidence of population stratification was revealed for the study population using the genomic control method [27], since the estimated inflation factor was near one (i.e., no inflation).

### Testing Incompatibility Effects

The overall best analysis model identified in the simulation study was used to code the incompatibility effect in the PE dataset. Given that father's genotype and clinical information was unknown, the logistic regression was used to analyze the incompatibility model. Whether or not to adjust for maternal and/or fetal effects and the method of coding the incompatibility was dependent on the results from the simulation study. In addition, we performed logistic regression to examine the relationship between affection status (PE [1] vs. control [0]) for each SNP genotype individually (mother and offspring). An additive genetic model (e.g., AA = 0, Aa = 1, aa = 2) was used to code each SNP where the homozygote corresponding to the allele with the minor allele frequency (MAF) was coded as 2.

Given that a large number of tests were conducted, correcting for multiple testing was necessary. The false discovery rate (FDR) was used to correct for multiple testing since this procedure is less conservative than the Bonferroni correction, and can account for the inter-marker correlation that may be present among markers genotyped within the same candidate gene or region [28].

## Results

### Simulated Data

**Figure 2:**
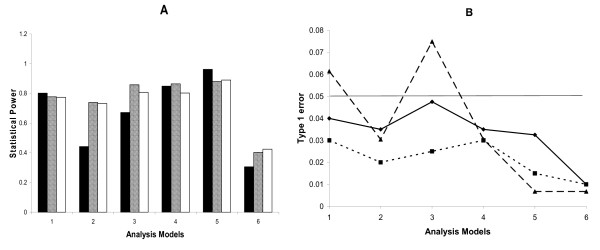
A) Power under each analysis model for the logistic model (black) and the log-linear model with equal number of families (diagonal lines) or individuals (white), and B) type I error rate under each analysis model for the logistic model (solid line), and the log-linear model with equal number of families (dotted line) or individuals (dashed line).

All of the analysis models were slightly conservative, since the type I error rates were less than the expected value of 0.05 in most cases. In addition, all of the models had comparable type I error rates in both the logistic and log-linear regression models with the exception of model 6, where the type I error rate was 50% less compared to model 5 (Figure 2b). Thus, the power of most of the models could be compared.

**Figure 3:**
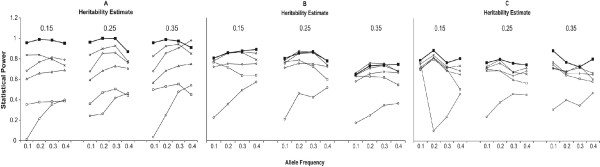
**The effect of allele frequency and heritability on the power for (A) the logistic model, (B) the log-linear model with equal number of families, and (C) log-linear model with equal number of individuals.** Model 1 (diamond), Model 2 (square), Model 3 (triangle), Model 4 (X), Model 5 (solid square), and Model 6 (circle).

Analysis model 5 (Table 2) was equally or more powerful than all of the other models in the logistic and log-linear regression unadjusted for maternal and fetal effects (Figure 2a). It is important to note that all of the other models are a subset of model 5, so model 5 is able to capture incompatibility effects regardless of the true generating model. There was little effect of heritability on the power for model 5 under the logistic regression, when averaged across the generating models and adjustment for maternal and fetal effects. For this model, the power increased by 4% for a heritability of 0.25 compared to a heritability of 0.15 followed by a slight decrease in power of 2% for a heritability of 0.35 compared to a heritability of 0.15. The effect of heritability on the power for model 5 using the log-linear model was similar, with the exception that there was a much larger decrease in power at a heritability of 0.35. For this model, the power decreased 2% for a heritability of 0.25 compared to a heritability of 0.15, and decreased by 10% for a heritability of 0.35 compared to a heritability of 0.15. For both the logistic and log-linear approaches, model 5 consistently performed similar to or better than all other models for all allele frequencies simulated (Figure 3). Although these results are locus specific it is important to note that the heritability estimates refer to the heritability of the underlying quantitative trait.

**Figure 4:**
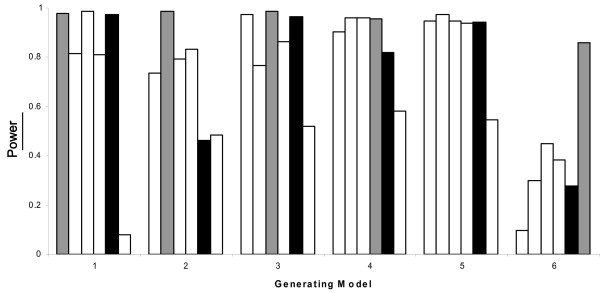
**Power of each analysis model under each generating model for the logistic model averaged across all allele frequencies and heritability estimates.** Models 1–6 are presented in order from left to right for each generating model. Model 5 (black bar) has similar power to the generating model (grey bar) in nearly all cases.

As expected, the power to detect a statistically significant incompatibility was the highest when the true underlying generating model was identical to the analysis model (Figure 4). This result was observed for both the logistic and log-linear regression models with the exception of model 6 for the logistic regression. This may be explained by the low type I error for analysis model 6 (Figure 2b). Although none of the models performed consistently well in all situations, analysis model 5 performed either equally well or slightly less powerfully compared to the true analysis models when averaged across all heritability values for all models except models 2 and 6 (Figure 4). Model 5 is also a reasonable 'default' model, because it encompasses all of the other models of incompatibility.

**Figure 5:**
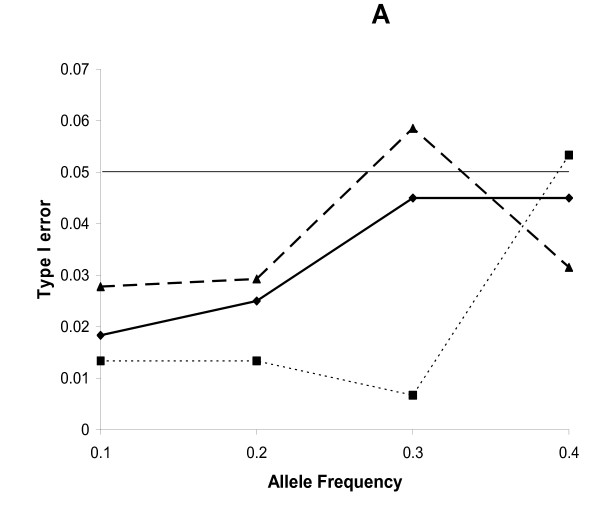
Type I error of logistic model (solid) and log-linear model with equal number of families (dotted) or individuals (dashed) as a function of allele frequency.

The trend in the type I error rate as a function of allele frequency differed between the logistic and log-linear regression (Figure 5). The type I error for the logistic regression in models unadjusted for maternal and fetal effects increased as the allele frequency increased. For the log-linear regression, when the number of individuals was the same, the trend was similar. However, when the number of families was the same, the type I error rate was more conservative for the log-linear regression compared to the logistic regression, except at the allele frequency of 0.4.

**Figure 6:**
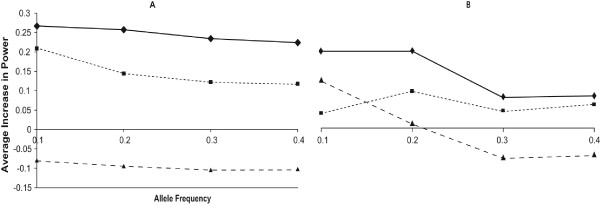
Average increase in power for the log-linear regression compared to the logistic regression with identical number of (A) families or (B) individuals for a heritability of 0.15 (solid), 0.25 (dotted), and 0.35 (dashed).

The relative power of the logistic regression and the log-linear regression depended on the heritability. For a heritability of 0.15 and 0.25, the power of the log-linear regression was greater than the power of the logistic regression when averaged over all of the analysis models by 25% and 15%, respectively. In contrast, for a heritability of 0.35, the power of the log-linear regression was less than the power of the logistic regression by 9% (Figure 6A). Overall, as the heritability increased, the relative advantage of the log-linear regression decreased compared to the logistic regression.

**Figure 7:**
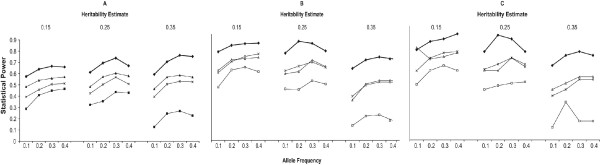
**The power of (A) the logistic model, and the log-linear model with equal number of (B) families or (C) individuals.** Models include incompatibility only (diamond), incompatibility plus maternal effects only (triangle), fetal effects only (X), or maternal and fetal effects (square).

Adjusting for maternal and fetal effects in addition to the incompatibility effect, by adding these effects into the analysis model, generally decreased power compared to adjusting for the incompatibility effect alone (Figure 7). The decrease in power depended on the heritability, and was the greatest for the heritability of 0.35, where adjustment for maternal and/or fetal effects reduced the power by as much as 50% for the logistic regression and 20% for the log-linear regression. Although separate maternal and fetal effects were not simulated, when we evaluated the simulated datasets for the presence of these effects, 80% of the datasets had either maternal or fetal effects, while 10% of the datasets had both effects. This is a limitation in the way simulations were conducted. We could not simulate incompatibility effects independent of maternal or fetal effects while keeping all the constraints mentioned above. However, we did evaluate type I error rates and confirmed that when maternal or fetal effects were not present, the type I error for detecting an incompatibility effect was not underestimated when adjusting for maternal and fetal effects in the logistic regression (data not shown). Similar results were found for the log-linear regression however, the log-linear regression model has slightly lower type I error rates for the adjusted models compared to the logistic regression. Overall, the log-linear regression appears to be more robust than the logistic regression to the adjustment for maternal or fetal effects, since the reduction in power is smaller.

### Pre-Eclampsia Data

To evaluate the PE data, we were limited to the logistic regression approach, since fathers were not recruited in this study. However, based on the results of the simulation study, we selected analysis model 5 to test the incompatibility effect, and we did not adjust for maternal and fetal effects. None of the SNPs in this study had a statistically significant incompatibility effect after correcting for multiple testing using the FDR approach and hence only nominal p-values are presented. However, this analysis revealed several interesting candidate SNPs with nominal p-values < 0.01 for the incompatibility effect (Table 3).

**Table 3:** Genes in which a SNP has a p-value < 0.01 for the maternal-fetal incompatibility model in the pre-eclampsia dataset

Gene Symbol	rs Number	Incompatibility model P-value	Additive Model P-value		% of Incompatibility		Odds Ratio^1^	95% CI^1^
			
			Maternal effects	Fetal effects	Cases	Controls		
LTA	2857713	0.0007	0.53	0.67	29.6	35.1	0.439	[0.273, 0.708]
IGF1R	28401726	0.0017	0.42	0.23	11.7	8.54	3.089	[1.530, 6.238]
VWF	216900	0.0037	0.15	0.86	28.9	17.9	2.104	[1.273, 3.480]
VWF	216321	0.0083	0.17	0.59	24.6	19.6	1.940	[1.185, 3.176]
COL4A2	2296849	0.0046	0.01	0.03	24.5	16.2	2.193	[1.274, 3.773]
COL4A2	421177	0.0054	0.02	0.22	32.2	23.2	1.952	[1.219, 3.125]
IGF1	5742620	0.0088	0.01	0.48	8	3.65	3.504	[1.371, 8.955]
IL2RA	10752175	0.0082	0.09	0.22	44.7	34.9	1.797	[1.163, 2.777]

^1^Model unadjusted for Maternal and Fetal Effects

SNP rs2857713 in the lyphotoxin alpha (*LTA*) gene showed the strongest evidence for association with PE in the incompatibility model (p = 0.0007). The marginal maternal and fetal effects were not statistically significant (Table 3), suggesting that the unadjusted model was appropriate. None of the remaining four SNPs evaluated in this gene showed evidence of association with PE for the incompatibility model, and none of the remaining SNPs were in LD with SNP rs2857713. The associated SNP is in exon 2 of *LT*A and encodes an amino acid change from cysteine to arginine at position 13 of the protein sequence. Interestingly, the incompatibility has a protective effect (OR = 0.439), where cases have a lower frequency of incompatibility compared to controls (Table 3). The greatest increase in risk occurs when both the mother and the offspring are heterozygous.

Two SNPs in the von Willebrand Factor (*VWF*) gene showed evidence for association with PE in the incompatibility model (Table 3). None of the remaining nine SNPs evaluated in this gene showed evidence of association with PE for the incompatibility model, except rs1800377 (p = 0.1099) in exon 12. However, the highest measure of LD between SNPs evaluated within this gene was 0.45. SNP rs216900 is in intron 37, whereas SNP rs216321 is a non-synonymous SNP in exon 20 causing an arginine to glutamine amino acid change in the protein sequence. Both SNPs showed an increased risk of PE for those with an incompatibility (Table 3). In particular, the genotype combination where the maternal genotype was 'AG' ('AG') and the offspring genotype was 'AA' ('AA') for SNP rs216900 (rs216321) showed the greatest increase in frequency for case compared to control maternal/offspring pairs.

Finally, the gene encoding for the alpha 2 chain of type IV collagen (*COL4A2*) also had two SNPs with suggestive evidence of association with PE for the incompatibility model including rs2296849 (p = 0.0046) and rs421177 (p = 0.0054). None of the remaining 7 SNPs evaluated in this gene showed evidence of association with PE for the incompatibility model, except COL4A2_633876793 (p = 0.0474). These two SNPs are both intronic polymorphisms in introns 37 and 38, respectively, of this gene. The two SNPs have moderate levels of LD between them, with r^2 ^of 0.62, and are located within 6 kb of each other. Both SNPs were more likely to have different (incompatible) genotypes for the mother/offspring pairs with PE compared to mother/offspring pairs without PE (Table 3). The genotype combination where the maternal genotype was 'CG' ('CG') and the offspring genotype was 'CC' ('CC') for SNP rs2296849 (rs421177) showed the greatest increase in frequency for case maternal/offspring pairs compared to control maternal/offspring pairs. Moderate maternal and fetal effects were observed for the SNPs in this gene, suggesting that it may potentially be important to control for these effects when evaluating the incompatibility model, although that was not done here because of the loss of power. None of the remaining genes (Table 3) had additional suggestive SNPs for the incompatibility model.

## Discussion

By conducting a comprehensive simulation study, we were able to identify a single analysis model with the best performance when the true underlying generating model is unknown. Model 5 consistently performed comparably or better than the alternative analytical models under all allele frequencies, heritability values, and in models adjusted and unadjusted for maternal and fetal effects. Model 5 incorporates all possible maternal/offspring genetic incompatibilities. Once an incompatibility effect has been detected, investigation of the proportion of mother/offspring pairs with specific genotype combinations (Table 1) may provide further insights into the biological mechanism of disease.

Our simulation study also showed that the performance of the logistic regression and the log-linear regression was similar, but dependent on the underlying conditions. The difference in power was relatively modest, approximately 10–20%, and the relative performance of the methods depended on heritability. More importantly, the regression methods differ in 1) the type of sample that must be recruited, and 2) the ability to test for maternal and fetal effects in addition to the incompatibility effects. In theory, estimating incompatibility effects can be biased when maternal and fetal effects are present, but are not accounted for. Likewise, when an incompatibility is present and not modeled explicitly, offspring and maternal effects, or both, can be biased [5]. Our observations indicated that the power to detect an incompatibility effect could be reduced by as much as 50% for the logistic regression versus a much smaller reduction in power for the log-linear regression. Log-linear regression can perhaps more efficiently handle the correlation between the maternal and fetal genotypes compared to the logistic regression. Thus, the considerations of study design and marginal maternal and fetal effects should play a greater role in determining which analytical approach to utilize.

Our analysis for incompatibility effects in the PE data set revealed several interesting candidate genes. *COL4A2 *is an extracellular matrix gene which encodes one of the six subunits of type IV collagen, the major structural component of basement membranes [29], and may be involved in tissue remodeling [30]. *COL4A2 *is a plausible candidate, since defects in the extracellular matrix due to polymorphisms in one of the subunits of type IV collagen, may be involved in the failure of transformation of uterine arteries that occurs in PE as well as other obstetrical complications, which some investigators attribute to abnormal trophoblast migration. In addition, there is some evidence to suggest that collagen is increased in PE, including a two-fold increase in expression level of collagen in the PE placenta [31], and decreased activity of cathepsin D activity in the PE umbilical cord, which may result in reduced collagen degradation and subsequent accumulation of collagen in the umbilical cord and uterine arteries [32]. Thus, polymorphisms in collagen that impact expression levels or degradation by cathepsin D could influence the risk of developing PE.

VWF, a large glycoprotein encoded by a gene on chromosome 12, is synthesized by vascular endothelial cells and circulates in human plasma. A possible mechanism of action for VWF is that abnormalities in VWF in fetal endothelial cells may interact with circulating maternal VWF during fetal trophoblast invasion of maternal spiral arteries and hence lead to the progression of PE. Although it is unclear how this interaction would lead to disease, this proposed mechanism is supported by the fact that altered patterns of VWF multimers are found to occur frequently in patients with thrombotic thrombocytopenic purpura in the acute and chronic stages, which shares some clinical and laboratory findings with PE including thrombocytopenia. In addition, although the sample sizes were small, Pottecher and coworkers [33] showed significantly increased plasma levels of VWF in women with PE compared to non-pregnant women (p = 0.009) or women with uncomplicated pregnancies (p = 0.037). Bergmann and colleagues [34] showed that alterations in the structure or biological function of VWF were not significantly associated with PE; however, they did not evaluate the role of VWF in the incompatibility model. In our analysis, the risk for PE is only elevated in the incompatibility model rather than independent effects from the maternal or offspring genotypes.

*LTA *was the most significant gene found to be associated with PE in the incompatibility model. LTA, also known as tumor necrosis factor-β (TNFβ), is a pro-inflammatory cytokine with a broad spectrum of immunological activities [35]. Circulating inflammatory cytokines such as TNFα have been implicated in the pathogenesis of PE [36-38]. In addition, the *LTA *gene lies in close proximity to *TNF*, the gene for tumor necrosis factor α [39-41]. Studies have shown that the plasma concentration of TNF*α *is significantly increased in PE patients. Although LTA has not been found to be associated with PE, it is possible that polymorphisms in the maternal and fetal LTA gene may decrease the circulating levels of LTA leading to a reduction in the inflammatory response, and a reduced risk for PE. This association offers a unique scenario where incompatible genotypes benefit the mother and fetus rather than causing harm.

The findings of this study highlight one important concept regarding the incompatibility model, which is that a genetic incompatibility between maternal and fetal genotypes can be beneficial. It is therefore somewhat unfortunate that the term "incompatibility" is used in this context since the negative connotation of the word implies that disease occurs only when the maternal and fetal genotypes are different. However, it may, in fact, be beneficial for the maternal and fetal genotypes to be different, resulting in positive selection, i.e., an excess of heterozygosity. Preference for different genotypes may be a mechanism to promote outcrossing due to the effects of dominance, since mating with close relatives will increase the chance of homozygous recessive genotype combinations. Alternatively, a preference for different genotypes may be an artifact of overdominance, where it is beneficial for the offspring to be heterozygous. Heterozygosity may be advantageous for several reasons such as increased variation in gene products resulting in improved chances to bind foreign proteins, or an increased chance of possessing rare alleles that infectious organisms have not yet developed resistance to.

One example of a locus where it is thought to be beneficial to be heterozygous is the Major Histocompatibility (MHC) locus. The increased heterozygosity at this locus may be a result of disassortative mating, where mates are preferentially selected to have different MHC alleles. This selection process is hypothesized to occur through the olfactory system, where individuals prefer the odor of people with different MHC alleles [42-44]. However, it is also possible that selection occurs at this locus through mechanisms other than mate choice. Females mating with males with the same MHC haplotype have increased fetal loss in humans [44-47] and primates [48], and increased chance of recurrent spontaneous abortions and unexplained infertility [49]. Thus, a fetus with similar genotypes to the mother is at increased risk of loss compared to a fetus with different genotypes than the mother. Haig [50] explored alternative models for these observations including gestational drive, where it is beneficial for the mother and fetus to be compatible, and a model of incompatibility. Both models could produce similar observations in the distribution of genotypes. Thus, although the mechanism of disease at this locus is incompletely understood, beneficial effects of genetic incompatibility remain a possibility.

Generalization of the study findings outside the scope of this work should be done with caution. We simulated genetic models based upon estimated model parameters for PE. Previous studies evaluating the heritability of the variance in liability of PE found that 35% of the variance is attributable to maternal effects, 20% to fetal effects, and 13% to the couple effect [25]. Therefore, we simulated genetic models with heritability between 0.15 and 0.35, since locus specific effects may not entirely account for all of the heritability for this disease. In addition, we simulated models with only an incompatibility effect, and without marginal maternal or fetal effects. This assumption could particularly impact the relative power of the models that are adjusted versus unadjusted for these effects. Finally, although we have identified compelling genetic candidates for association with PE with the incompatibility model, none of these findings were statistically significant after correcting for multiple testing. Given the frequency of conflicting or erroneous reports of genetic association, these findings should be interpreted with caution unless they are replicated with similar large studies of PE.

There are several limitations of the study. We were unable to simulate incompatibility effects independent of maternal or fetal effects and hence the power to detect an incompatibility effect was significantly reduced in the adjusted models compared to the unadjusted models. However the log-linear models were more robust to this adjustment. In addition, we only looked at minor allele frequencies ranging from 0.1 to 0.4. Model 5 may have performed equally well to the true analysis models due to similar frequencies of the incompatible genotype combinations to the true analysis model. This is a characteristic of model 5 as it incorporates all possible incompatible genotype combinations by the other models, and hence under similar allele frequencies, the proportions may be similar. Specifically model 5 may have had the best performance compared to model 6 since the number of incompatible genotype combinations was greater in model 5 compared to model 6 and the genotype combinations were dominated by AA, Aa genotype combinations. This may also explain the relative performance of models 1 and 4 compared to model 5; however, it seems unrealistic to simulate conditions where the MAF is > 0.4 which is very rare in populations [51]. Nevertheless, we have shown that model 5, which includes all possible combinations of incompatible genotypes, performs as well as the simulated model in all datasets, suggesting that if the true underlying model is unknown, model 5 has the best power to detect the incompatibility.

## Conclusion

In summary, we have identified a single incompatibility model that has optimal properties in terms of power and type I error compared to the alternative models under the specific conditions simulated. Our results also indicate that the relative performance of the logistic regression and log-linear regression approaches are similar, and that issues of study design including the ability to recruit fathers and the presence of marginal maternal and fetal effects are the most important considerations. We also identified three genes, *COL4A2*, *VWF*, and *LTA *which may have a significant functional role in the progression of PE under the incompatibility model, although none of these findings were statistically significant after correcting for multiple testing using the FDR. Little is known about the biological mechanism though which maternal and fetal incompatibility of these genes may have an adverse or protective effect on the mother. Future studies both epidemiological and molecular are needed to confirm the biological mechanism of the incompatibility effect among these genes. However, these findings will be helpful in the design and interpretation of future studies of incompatibility effects, particularly studies of PE.

## Competing interests

The authors declare that they have no competing interests.

## Authors' contributions

KABG and NP contributed to the conception and design of the methodological study, carried out the statistical analysis, participated in the interpretation of data, and drafted the manuscript. GT and HK designed and coordinated the molecular genetic studies for the PE dataset, contributed to the interpretation of data, and critically reviewed the manuscript. RR conceived of and directed the PE study, contributed to the data interpretation, and critically reviewed the manuscript. JKN and RG participated in patient recruitment and validated the clinical information for the PE study. All authors read and approved the final manuscript.

## APPENDIX

Equations

(1) Logit [Y = 1] = -β + β_1_*I (G_m_, G_off_)

(2) Logit [Y = 1] = β + β_1_*I_(Gm) _+ β_2_*I_(Goff) _+ β_3_*I_(Gm, Goff)_

(3) Logit [Y = 1] = β + β_1_*I_(Gm) _+ β_3_* I_(Gm, Goff)_

(4) Logit [Y = 1] = β + β_2_*I_(Goff) _+ β_3_* I_(Gm, Goff)_

For the following equations, the variables are defined in Table 4.

**Table 4:** Case parent/offspring triads and expected triad counts for analysis model 3 using the log-linear regression

Cell	Mating Type	Mother/Father Genotype	Offspring Genotype	Expected Counts
	J	G_m_, G_f_	G_off_	ω_i_
1	1	A/A, A/A	A/A	ηρδ_1_
2	2	A/A, A/a	A/A	ηρδ_2_
3	2	A/a, A/A	A/a	ηρδ_2_
4	2	A/a, A/A	A/A	ηρδ_2_
5	2	A/a, A/A	A/a	ηρδ_2_
6	3	A/A, a/a	A/a	ηρδ_3_
7	3	a/a, A/A	A/a	μρδ_3_
8	4	A/a, A/a	A/A	ηρδ_4_
9	4	A/a, A/a	A/a	2ηρδ_4_
10	4	A/a, A/a	a/a	ηδ_4_
11	5	A/a, a/a	A/a	ηρδ_5_
12	5	A/a, a/a	a/a	ηδ_5_
13	5	a/a, A/a	A/a	μρδ_5_
14	5	a/a, A/a	a/a	δ_5_
15	6	a/a, a/a	a/a	δ_6_

Where η is the relative risk when the variant allele/s is present in the mother, ρ is the relative risk when the variant allele/s is present in the child, μ is the relative risk associated with the maternal-fetal genotype incompatibility and δ_j _is the mating type where j = 1 to 6.

(5) ln(ω_i_) = ln (δj) + ln (μ)I_(Gm, Goff) _+ ln(2)I_(Gm = Gf = Goff)_

(6) ln(ω_i_) = ln (δj) + ln (μ)I_(Gm, Goff) _+ ln(η)I_(Gm) _+ ln(ρ)I_(Goff)_+ ln(2)I_(Gm = Gf = Goff)_

(7) ln(ω_i_) = ln (δj) + ln (μ)I_(Gm, Goff) _+ ln(ρ)I_(Goff) _+ ln(2)I_(Gm = Gf = Goff)_

(8) ln(ω_i_) = ln (δj) + ln (μ)I_(Gm, Goff)+ _ln(η)I_(Goff) _+ ln(2)I_(Gm = Gf = Goff)_

## Pre-publication history

The pre-publication history for this paper can be accessed here:


